# Correction to “TMC-1: Probing the Onset of Chemical Complexity in Space”

**DOI:** 10.1021/acsearthspacechem.6c00222

**Published:** 2026-07-06

**Authors:** Marcelino Agúndez, José Cernicharo

An error occurred in the preparation of Table 1 upon conversion from an ascii file to the three-column LaTeX table in which the last five molecules (all S-bearing) were omitted. Table 1 therefore contained only 183 molecules, while the inventory of molecules detected in TMC-1 is 188. Here we report the five missing S-bearing molecules with the corresponding column densities and references.

**Table 1:** Five Missing S-Bearing Molecules Detected in TMC-1 in Table 1 of the Original Article^a^

molecule	*N* (cm^–2^)	ref
NC_3_S	1.4 × 10^11^	^1^
C_4_S	3.8 × 10^10^	^2^
HC_4_S	9.5 × 10^10^	^3^
C_5_S	3.0 × 10^10^	^2^
NCCHCS	1.2 × 10^11^	^4^

a Column densities can be converted to abundances relative to H_2_ by dividing by 10^22^ cm^–2^ (ref 5).
